# Alterations of cerebral perfusion and functional brain connectivity in medication‐naïve male adults with attention‐deficit/hyperactivity disorder

**DOI:** 10.1111/cns.13185

**Published:** 2019-06-23

**Authors:** Ya‐Wen Tan, Lu Liu, Yan‐Fei Wang, Hai‐Mei Li, Mei‐Rong Pan, Meng‐Jie Zhao, Fang Huang, Yu‐Feng Wang, Yong He, Xu‐Hong Liao, Qiu‐Jin Qian

**Affiliations:** ^1^ Peking University Sixth Hospital/Institute of Mental Health Beijing China; ^2^ National Clinical Research Center for Mental Disorders (Peking University Sixth Hospital) & the NHC Key Laboratory of Mental Health (Peking University) Beijing China; ^3^ State Key Laboratory of Cognitive Neuroscience and Learning Beijing Normal University Beijing China; ^4^ Beijing Key Laboratory of Brain Imaging and Connectomics Beijing Normal University Beijing China; ^5^ School of Systems Science Beijing Normal University Beijing China

**Keywords:** ADHD, cerebral blood flow, functional connectivity, resting‐state fMRI

## Abstract

**Aims:**

Functional brain abnormalities, including altered cerebral perfusion and functional connectivities, have been illustrated in adults with attention‐deficit/hyperactivity disorder (aADHD). The present study attempted to explore the alterations of cerebral blood flow (CBF) and resting‐state functional connectivity (RSFC) simultaneously to understand the neural mechanisms for adults with ADHD comprehensively.

**Methods:**

Resting‐state arterial spin labeling (ASL) and blood oxygenation level‐dependent (BOLD) magnetic resonance imaging (MRI) data were acquired for 69 male aADHD and 69 matched healthy controls (HCs). The altered CBFs associated with aADHD were explored based on both categorical (aADHD vs HCs) and dimensional (correlation with aADHD core symptoms) perspectives. Then, the seed‐based RSFC analyses were developed for the regions showing significant alterations of CBF.

**Results:**

Significantly decreased CBF in the large‐scale resting‐state networks regions (eg, ventral attentional network, somatomotor network, limbic network) and subcortical regions was indicated in aADHD compared with HCs. The correlation analyses indicated that the hypoperfusion in left putamen/global pallidum and left amygdala/hippocampus was correlated with ADHD inattentive and total symptoms, respectively. Further, weaker negative functional connectivity between left amygdala and bilateral supplementary motor area, bilateral superior frontal gyrus, and left medial frontal gyrus was found in adults with ADHD.

**Conclusion:**

The present findings suggested alterations of both cerebral perfusion and functional connectivity for the left amygdala in aADHD. The combination of CBF and RSFCs may help to interpret the neuropathogenesis of ADHD more comprehensively.

## 1 INTRODUCTION

Attention‐deficit/hyperactivity disorder (ADHD) is described by developmentally inappropriate symptoms of inattention, hyperactivity, and impulsivity,^1^ which is childhood‐onset neurodevelopment disorders. The estimated worldwide prevalence of adult ADHD (aADHD) is 2.5%.^2^ Emotional dysregulation and substance abuse are important associated features and comorbidities in aADHD, leading to significant personal and societal costs.^3^, ^4^

However, the neurobiology mechanism of ADHD has been unclear till now. Accumulating evidences suggested brain structural and functional brain abnormality in both children and adults with ADHD. Abnormalities of the regions and networks have been found in ADHD using both structural and functional measures by multiple modalities, including structural magnetic resonance imaging (MRI),^5^ diffusion tensor imaging (DTI),^6^ and functional MRI (fMRI).^7^, ^8^ For fMRI, resting‐state functional connectivity (RSFC) has been widely used as an important feature reflecting brain function and suggested multiple networks possibly involved in the neural mechanism of ADHD, such as default mode network, frontoparietal network, ventral attention network, dorsal attention network, and so on.^7^ Besides, cerebral blood flow (CBF), as a physiology parameter closely related to cerebral metabolism,^9^ has received much attention in recent years. CBF is defined as the blood volume that delivers to per unit mass per unit time in brain tissue.^10^ CBF is of vital importance for brain function. It ensures the proper and enough delivery of oxygen and energy substance for brain and transports the metabolic waste away.^11^

In previous studies, positron emission tomography (PET) and single photon emission computerized tomography (SPECT) have been widely used to identify CBF abnormalities in brain regions in ADHD. Hypoperfusion in the striatum has been consistently demonstrated in previous studies.^12^, ^13^ In addition, abnormal perfusion in frontal, temporal, and cerebellar regions^12^, ^13^, ^14^, ^15^ was often reported in spite of conflicts. However, as invasive procedures, PET and SPECT still take a long time before acquisition with low spatial resolution.^16^ In the past decade, the arterial spin labeling (ASL) MRI is extensively used to rapidly quantify CBF as a noninvasive technique by an endogenous contrast.^17^, ^18^, ^19^ The CBF measured by ASL‐MRI was considered to be closely coupled with glucose metabolism measured by PET.^20^ O'Gorman et al^21^ found increased regional cerebral perfusion in adults with ADHD in the left caudate nucleus, frontal and parietal regions by using continuous arterial spin labeling (CASL) technique, which could be normalized by stimulant treatment. A recent pharmacological magnetic resonance imaging (phMRI) study supported the above finding in some extent, demonstrating decreased CBF in extent cortical areas for adults with ADHD after an acute challenge with methylphenidate (0.5 mg/kg).^22^

As a physiological parameter of brain metabolism, CBF is also closely related to neuronal activity.^23^ According to the neurovascular coupling hypothesis, increased CBF may be coupled with higher degree of functional brain connectivity.^10^ The BOLD signal is considered to measure the changes in neuronal activity‐related blood oxygenation and reflects the intrinsic functional activation of the brain.^24^ The seed‐based functional connectivity detected by BOLD fMRI measures the temporal correlations of low‐frequency fluctuations. It is widely used because of its inherent simplicity, sensitivity, and ease of interpretation.^24^ Different aspects of resting‐state neuronal activity may be captured by CBF and BOLD signal. Investigating the collaboration of ASL and BOLD could provide a more comprehensive picture on the physiological and pathophysiological mechanisms of the brain.^23^ Recently, Liang et al^25^ investigated the relationship between CBF and RSFC of the whole brain in healthy adults and reported a tight relationship between CBF and the functional connectivity of the brain in the DMN and executive control network. In addition, altered coupling between CBF and functional connectivity has been illustrated for several neuropsychiatric disorders, such as schizophrenia,^10^ mild cognitive impairment,^26^ and mood disorders.^27^ However, further research was still needed to investigate the correlation between CBF and functional connectivity in patients with ADHD.

In the current study, we attempted to investigate the alterations in CBF as well as seed‐based functional connectivity in male adults with ADHD by using ASL and BOLD fMRI. Firstly, we investigated the brain regions with altered cerebral perfusion in male aADHD based on both categorical (male aADHD vs male age‐matched healthy controls) and dimensional (correlation with ADHD core symptoms) perspectives. Following the identification of ADHD‐related alteration of CBF, the potential functional connectivity disruptions for these CBF‐altered regions were further evaluated by seed‐based FC analysis based on resting‐state BOLD fMRI. To exclude the potential confounding influence of gender^28^ on the results, our current study only included male subjects to reduce the sample heterogeneity.

## 2 MATERIALS AND METHODS

### 2.1 Participants

A total of 138 male adults were recruited for the present study, including 69 patients with ADHD recruited from clinics of Peking University Sixth Hospital and 69 healthy controls matched for age and intelligence quotient (IQ). The recruitment procedure was shown in a flowchart in detail (see Figure S1). All the participants signed the informed consent, and the current study was approved by the Research Ethics Review Board of Peking University Sixth Hospital.

All the participants were interviewed and underwent diagnosis by a qualified psychiatrist, screening for any potential comorbidities according to exclusion criteria using the Structured Clinical Interview for DSM‐IV Axis I Disorders (SCID‐I).^29^ To confirm the diagnosis of ADHD, Conner's Adult ADHD Diagnostic Interview for DSM‐IV^30^ was also interviewed. Full‐scale IQ measurements were made using the Wechsler Adult Intelligence Scale‐Third Edition. Furthermore, to the evaluation of the severity of ADHD symptoms, the ADHD Rating Scale‐IV (ADHD RS‐IV)^31^was completed by the participants.

The inclusion criteria were as follows: (a) aged above 18 years; (b) right hand dominant; (c) no history of severe physical disease; (d) no current diagnosis of schizophrenia, severe major depression, clinically significant panic disorder, bipolar disorder, or mental retardation; (e) a full‐scale IQ above 90; and (f) psychoactive medicine naïve. A previous history or current diagnosis of psychiatric disorders, as evidenced in the SCID‐I assessment, or neurological disorders resulted in exclusion from the HC groups. Eight ADHD patients had the history of major depressive disorder, while three patients suffered the general anxiety disorder. In addition, if the head movements are more than 3 mm translation or three degrees of rotation in any direction, the subjects will be excluded. In current study, no subject was excluded based on the head movements. The demographic and clinical data for participants are given in Table 1 in detail.

**Table 1:** Demographic and clinical information

Variables	ADHD (n = 69) (mean ± SD)	HC (n = 69) (mean ± SD)	*T*‐value	*P*‐value
Age (y)	26.5 ± 3.9	26.3 ± 3.9	0.24	0.810
Full‐scale IQ scores	120.72 ± 7.49	121.72 ± 7.92	−0.76	0.447
ADHD subtypes (inattentive/combined)	54/15	–	–	–
ADHD core symptoms				
Total	46.49 ± 7.60	25.60 ± 5.84	15.57	<0.0001*
Inattention	27.16 ± 4.27	13.40 ± 3.29	17.62	<0.0001*
Hyperactivity/impulsivity	19.46 ± 5.26	12.19 ± 3.11	8.53	<0.0001*
Mean FD (mm)	0.100 ± 0.05	0.108 ± 0.07	−0.73	0.466

Abbreviations: ADHD, attention‐deficit/hyperactivity disorder; FD, framewise displacement; HC, healthy control.

* The *P*‐value was obtained by the two‐sample two‐tailed *t* test.

### 2.2 Image acquisition

All MRI images were acquired using a 3.0‐Tesla MR system (General Electric; Discovery MR750) in the Center for Neuroimaging in Peking University Sixth Hospital. High‐resolution 3D T1‐weighted anatomical images were acquired using a three‐dimensional fast spoiled gradient‐recalled (3D‐FSPGR) sequence with the following parameters: repetition time (TR) = 6.66 ms; echo time (TE) = 2.93 ms; inversion time (TI) = 450 ms; flip angle (FA) = 8°; field of view (FOV) = 256 × 256 mm; matrix = 256 × 256; slice thickness = 1.0 mm with no gap, 180 sagittal slices. The resting‐state perfusion imaging was performed using a pseudo‐continuous ASL (pcASL) sequence with a 3D fast spin‐echo acquisition and background suppression: TR/TE = 4781/11.12 ms; postlabel delays = 1525 ms; spiral in readout of 12 arms with 512 sample points; FOV = 220 × 220 mm; matrix = 128 × 128; FA = 111°; slice thickness = 3 mm, no gap; 45 axial slices; and number of excitation = 3. Resting‐state BOLD images were acquired using a gradient‐echo single‐shot echo planar imaging (GRE‐SS‐EPI) sequence with the following parameters: TR/TE = 2000/30 ms; FA = 90°, slice thickness = 3.2 mm with no gap; matrix = 64 × 64; FOV = 220 × 220 mm; 43 axial slices and 240 volumes. During the scans, participants were instructed to relax with their eyes closed, stay still, and think of nothing in particular without falling asleep.

### 2.3 fMRI data preprocessing

Functional MRI data were preprocessed using the Data Processing & Analysis for (Resting‐State) Brain Imaging (DPABI).^32^ Firstly, the first 10 volumes of each participant were discarded. Next, we performed slice timing correction to correct the remaining volumes for the acquisition time delay and realignment for correcting head motion across volumes. The realigned functional data were spatially normalized by a nonlinear registration to the EPI template in MNI standard space^33^ and resampled to 3 mm isotropic voxels. The functional data were spatially smoothed with a 6 mm full‐width at half maximum (FWHM) Gaussian kernel. Linear detrending of the time series was conducted. Subsequently, we performed nuisance signal regression (including Friston's 24 head motion parameters, global mean signal, white matter, and cerebrospinal fluid signals) to reduce the confounding artifacts of head motion and physiological noises (ie, cardiac and respiratory fluctuations) during the resting state. Temporal bandpass filtering (0.01‐0.1 Hz) was conducted. Furthermore, data were preprocessed with scrubbing for validation (See Validation Analysis2.2), and the results are shown in the Supplementary Materials.

### 2.4 CBF quantification and statistical analysis

Generally, ASL imaging data consist of a control image and a label image. The quantitative CBF images were calculated by using a single‐compartment kinetic model^10^ by using ASLtbx.^34^ Then, SPM12 software (http://www.fil.ion.ucl.ac.uk/spm) was used to further process the CBF data. First, for each subject the T1 image was coregistered to the perfusion image, which was aligned with the corresponding CBF image. Next, the coregistered T1 image was segmented into gray matter, white matter, and cerebrospinal fluid. The CBF map was normalized into the MNI space based on the transform parameters that were estimated during the segmentation of the T1 image and further resampled to 3 × 3 × 3 mm^3^. Finally, the normalized CBF images were smoothed with 6 mm FWHM. CBF analysis was conducted within the gray matter mask without the cerebellum (*N*_voxel_ = 45 381). For each participant, the spatially normalized CBF images were transformed to *z*‐score by subtracting the mean and then being divided by standard deviation across all voxels in the gray matter mask for further statistical analysis. To identify ADHD‐related alterations of CBF, we conducted categorical and dimensional analyses sequentially. Firstly, between‐group comparisons were assessed by using an independent two‐sample *t* test with age as covariates between the ADHD and HC groups. The significance of the statistical maps was corrected for multiple comparisons to a significant level of *P* < 0.05 through a permutation test (parameters: 10 000 permutations, individual voxel *P* < 0.01, cluster size >509). The statistical nonparametric mapping (SNPM) toolbox provides an extensible framework for voxel level nonparametric permutation tests of functional neuroimaging experiments with independent observation. It is a commonly used approach of an initial cluster‐forming thresholding, with generally better sensitivity.^35^, ^36^

Secondly, the association between the altered CBFs and the severity of ADHD symptoms was investigated. Correlation analyses were performed between CBF *z*‐value of brain regions showing significant between‐group differences and ADHD rating scale scores (inattention, hyperactivity/impulsivity, and total scores) in the ADHD group. We performed the voxel‐wise correlation within the brain regions showing significant intergroup differences. The statistical level of significance was corrected for multiple comparisons to a significant level of *P* < 0.05 through a nonparametric permutation test (parameters: 10 000 permutations, individual voxel *P* < 0.01, cluster size >28) using the SNPM toolbox.

### 2.5 Resting‐state functional connectivity analysis and statistical analysis

In order to evaluate the potential functional connectivity disruptions in the ADHD‐related CBF‐altered regions based on both categorical and dimensional perspectives, the seed‐based functional connectivity analysis was conducted. The seed regions of interest (ROIs) were defined as the spatial overlapping areas between the clusters showing significant CBF alterations and the Anatomical Automatic Labeling (AAL) template.^37^ For each ROI, the functional connectivity maps were calculated by Pearson's correlation coefficients between the mean time series of this ROI and the time series of each voxel in the gray matter for each participant.^38^ The Fisher's *z*‐transformation was conducted to each functional connectivity map.

Within each group of adults with ADHD or the HC, a one‐sample *t* test was performed to individual *z*‐score maps for each ROI, with age and mean framewise displacement (mFD) as covariates. The statistical level of significance was corrected for multiple comparisons to a significant level of *P* < 0.05 through a nonparametric permutation test (parameters: 10 000 permutations, individual voxel *P* < 0.01, cluster size >349) using SNPM toolbox.

The between‐group comparisons (ADHD vs HC) were assessed by using an independent two‐sample *t* test with age and mFD as covariates. For each seed ROI, functional connectivity comparison was conducted within the brain mask showing significant correlations with the seed in either group. The statistical level of significance was corrected for multiple comparisons to a significant level of *P* < 0.05 through a permutation test (parameters: 10 000 permutations, individual voxel *P* < 0.01, cluster size >231) using SNPM toolbox. The association between the abnormal functional connectivity and the severity of ADHD symptoms was further investigated. We extracted the amygdala‐based rsFC of the brain regions with abnormal functional connectivity. Correlation analyses were performed between amygdala‐based rsFC *z*‐value of brain regions with abnormal functional connectivity and ADHD rating scale scores (inattention, hyperactivity/impulsivity, and total scores) in the ADHD group. The statistical level of significance was a significant level of *P* < 0.05.

### 2.6 Validation analysis

The scrubbing procedure was further conducted during the fMRI data preprocessing to reduce the potential confounding influence of head motion on results. During the scrubbing process, volume with a FD > 0.5 mm would be scrubbed. And then, its prior one and next two volumes would be removed from this participant. In addition, 11 patients with comorbidities (eight patients with the previous history of major depressive disorder and three patients with current diagnosis of general anxiety disorder) were further excluded to verify the main results.

## 3 RESULTS

The main results were reported using the fMRI data without scrubbing in all 138 participants. The main findings remained almost unchanged when performing data scrubbing or excluding comorbidities (Figures S2‐S7).

### 3.1 Spatial distribution of CBF

**Figure 1:**
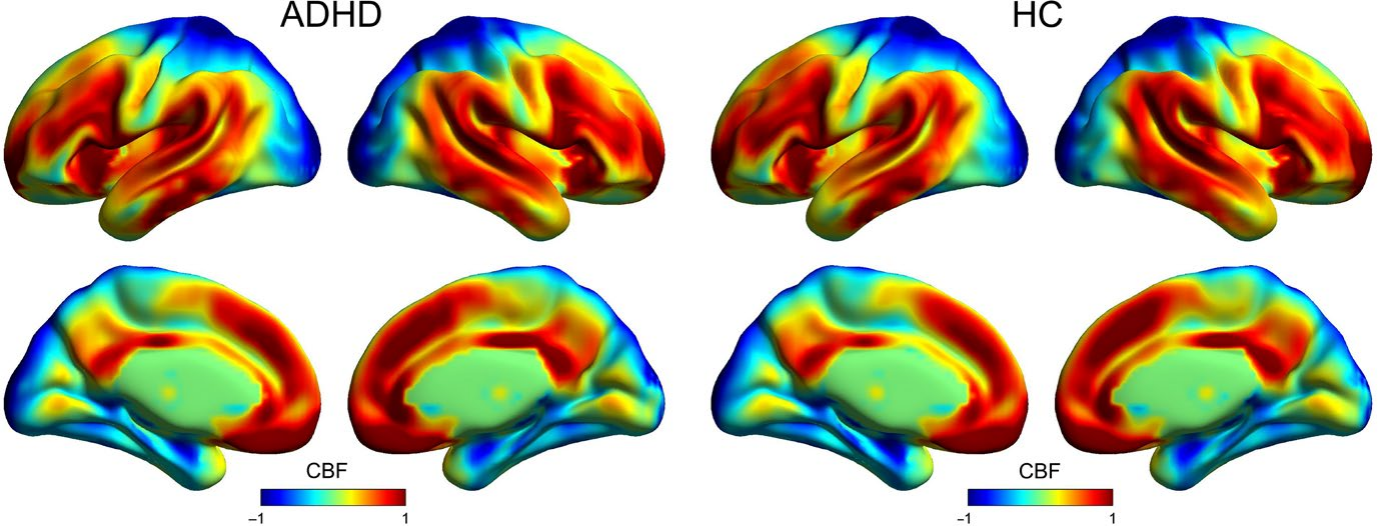
Spatial distribution maps of CBF at the group level. For each group (ie, ADHD or HC), the individual CBF maps were normalized to *z*‐scores and then averaged across subjects to generate a group‐level map. ADHD, attention‐deficit/hyperactivity disorder; CBF, cerebral blood flow; HC, healthy controls

Attention‐deficit/hyperactivity disorder patients and HCs showed similar spatial patterns in CBF (Figure 1). At the group level, brain regions with higher CBF were mainly located in the bilateral posterior cingulate cortex/precuneus, medial prefrontal cortex (PFC), anterior cingulate cortex, lateral temporal, and parietal cortices, regardless of the group considered. Most of the regions comprise the default mode network regions (DMN). In addition, regions located in the insula, lateral PFC, sensorimotor also showed high CBF in the both groups.

### 3.2 CBF changes in ADHD

**Figure 2:**
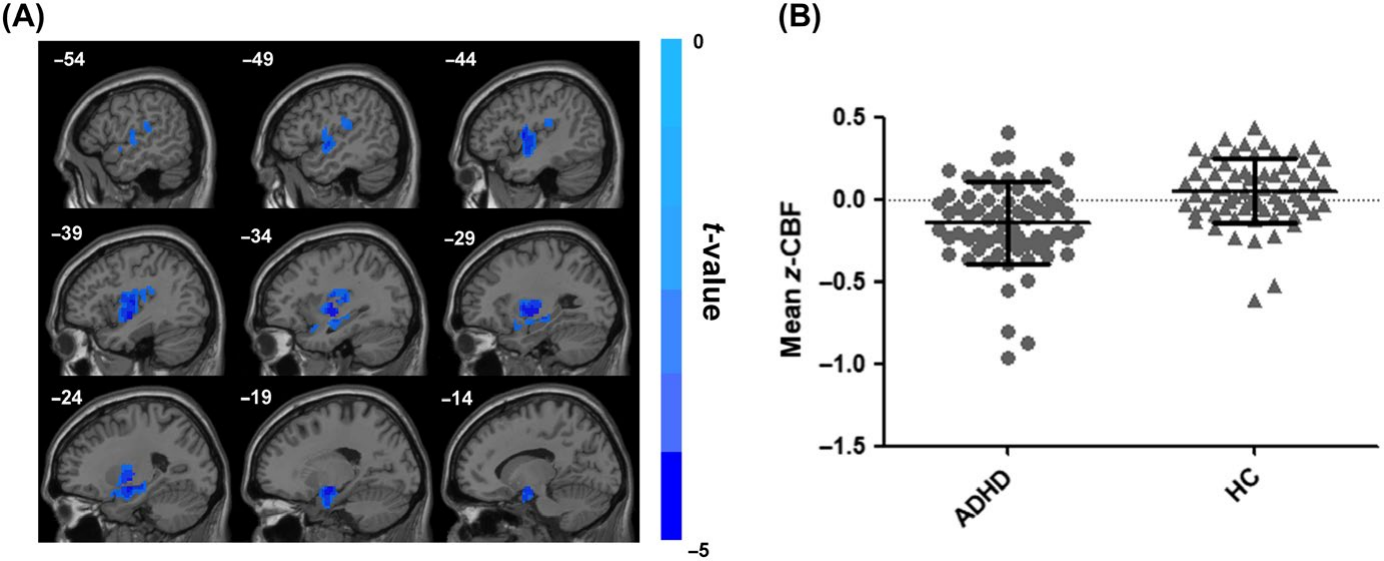
Group differences in CBF between ADHD patients and healthy controls. The independent two‐sample *t* test was conducted between the ADHD group and the healthy control group. All results were corrected for multiple comparisons to a significant level of *P* < 0.05 through a nonparametric permutation test (10 000 permutations, individual voxel *P* < 0.01) using SNPM toolbox. The cold colors denote significantly decreased CBF in the ADHD patients. ADHD, attention‐deficit/hyperactivity disorder; CBF, cerebral blood flow; HC, healthy controls

Between‐group comparison revealed significantly lower CBF in the ADHD group, primarily in the left hemisphere. The regions included insula, orbital frontal cortex, putamen, pallidum, amygdala, supramarginal gyrus, rolandic operculum, temporal gyrus, hippocampus, parahippocampal gyrus, and olfactory gyrus (family‐wise error, FWE corrected *P* < 0.05; Figure 2). These hypoperfusion regions mainly located in the somatomotor network, ventral attention network, limbic network, and subcortical regions (Table S1). No significantly increased CBF was found in ADHD group.

### 3.3 Correlation between CBF and ADHD core symptoms

**Figure 3:**
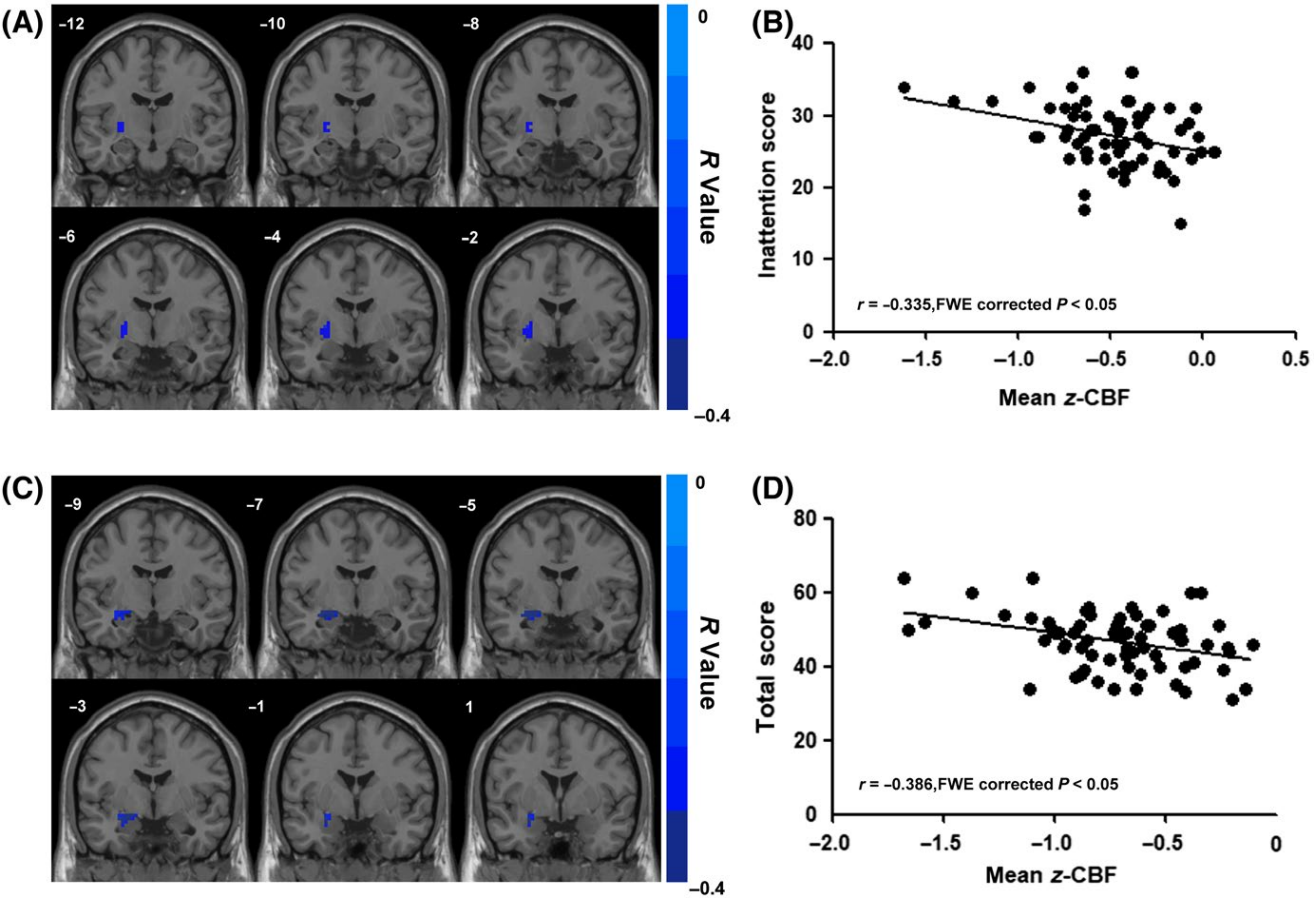
Correlation between CBF of regions showing between‐group differences (ADHD vs HC) and ADHD core symptoms. The correlation analysis was performed within ADHD patients with inattention, hyperactivity/impulsivity, and total symptom scores measured by ADHD RS‐IV. All results were corrected for multiple comparisons to a significant level of *P* < 0.05 through a nonparametric permutation test (10 000 permutations, individual voxel *P* < 0.01). A, The correlation between the left putamen/global pallidum z‐CBF and inattention scores in male aADHD. B, Scatter plot of correlation between the mean z‐CBF of left putamen/global pallidum and inattention scores in male aADHD. C, The correlation between the left amygdala/hippocampus z‐CBF and inattention scores in male aADHD. D, Scatter plot of correlation between the mean z‐CBF of left amygdala/hippocampus and total scores in male aADHD. ADHD, attention‐deficit/hyperactivity disorder; CBF, cerebral blood flow; HC, healthy controls

For the brain regions showing group‐difference CBFs, we further calculated the correlation between the altered CBF and the severity of ADHD core symptoms. In adults with ADHD, a significant negative correlation existed between the CBF in the left putamen/global pallidum and the inattention score (*r* = −0.335, FWE corrected *P* < 0.05; Figure 3A,B). In addition, we found a significant negative correlation between the CBF in the left amygdala/hippocampus and total score (*r* = −0.386, FWE corrected *P* < 0.05) (Figure 3C,D). We did not find any significant correlation for the hyperactivity/impulsivity score.

### 3.4 Resting‐state functional connectivity changes in ADHD

In our current study, ADHD‐related hypoperfusion regions were defined based on both categorical and dimensional perspectives which might help us to illustrate the cautious ADHD‐related abnormalities. Eventually, four brain regions, including putamen, pallidum, amygdala, and hippocampus, were used for seed‐based functional connectivity analysis (Table 2). The functional connectivity maps of these four ROIs were estimated by using BOLD fMRI, separately. Adults with ADHD exhibited the similar functional connectivity patterns with those of the HCs. For instance, a significantly positive functional connectivity with the left amygdala was shown in the partial cortex, temporal lobe, and limbic lobes, while a significant negative functional connectivity with the left amygdala was located in the occipital gyrus, temporal lobe, and PFC (Figure S2). Furthermore, the regions with significant positive or negative correlations with each ROI in two groups were combined to generate as masks for between‐group analysis, separately.

**Table 2:** Four brain regions used for seed‐based functional connectivity analyses

Area	L/R	Cluster size (number of voxels)
Amygdala	L	28
Hippocampus	L	12
Putamen	L	26
Pallidum	L	7

Note

The clusters with significant CBF alterations from both categorical and dimensional analyses were defined as the regions of interest (ROIs) which were extracted by overlapping the AAL template.

**Figure 4:**
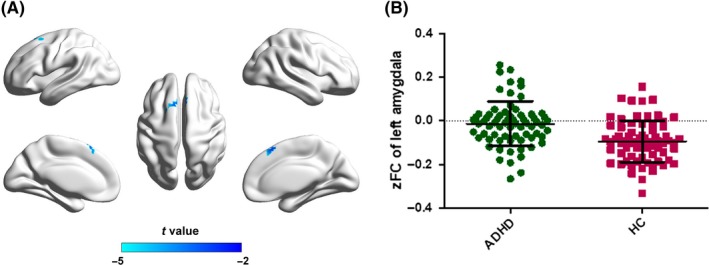
Between‐group comparisons for the seed‐based functional connectivity with the seeds of left amygdala. The independent two‐sample *t* test was conducted between the ADHD group and the healthy control group. All results were corrected for multiple comparisons to a significant level of *P* < 0.05 through a nonparametric permutation test (10 000 permutations, individual voxel *P* < 0.01, cluster size >231) using the SNPM toolbox. zFC, *z*‐value of functional connectivity. ADHD, attention‐deficit/hyperactivity disorder; CBF, cerebral blood flow; HC, healthy controls

Two‐sample *t* tests were performed to investigate the differences in functional connectivity of the ROIs between adults with ADHD and HCs. For the left amygdala ROI, significant decrease functional connectivity was observed in ADHD group primarily involving the bilateral supplementary motor area, bilateral superior frontal gyrus, and left medial frontal gyrus (FWE corrected *P* < 0.05) within the negative functional connectivity mask (Figure 4). No significantly different regions were found within the positive connectivity mask as well as connectivity masks of the other three ROIs. Correlation analyses between altered functional connectivity and ADHD core symptoms did not indicate any significant results (Table S2).

## 4 DISCUSSION

In this study, we investigated the CBF changes in the gray matter and the potential functional connectivity disruptions for the CBF‐altered regions in male adults with ADHD by combining pcASL and BOLD fMRI techniques. Firstly, compared with healthy controls, adults with ADHD showed significantly reduced perfusion in the regions involving in somatomotor network, ventral attention network, limbic network, and subcortical regions. Further analyses from dimensional perspective indicated significant negative correlation between the CBF in left amygdala/hippocampus and total symptoms, the left putamen/global pallidum, and inattention score, respectively. When further evaluating the potential changes in seed‐based functional connectivity for these hypoperfusion regions, weaker negative connectivities were found between left amygdala and the supplementary motor area, superior frontal gyrus, and left medial frontal gyrus.

### 4.1 Hypoperfusion of CBF in ADHD

Significantly reduced perfusion has been consistently reported in patients with ADHD in the somatomotor network, ventral attentional network, and limbic network in early PET and SPECT studies.^13^, ^14^, ^15^, ^39^ The hypoperfused regions in the frontoparietal network (eg, inferior frontal cortex and insula) observed in our study also showed abnormalities in structural and functional aspects by other MRI studies.^5^, ^40^, ^41^ Using CASL data, O'Gorman et al^21^ found that regional CBF increased in the adults with ADHD in the regions of left caudate nucleus, frontal and parietal cortices, indicating the abnormality of frontoparietal network involved in the neurobiological mechanism of ADHD. Although taking consideration of the same regions, the direction of the perfusion among the nonmedicated aADHD in our study differs to that in the O'Gorman's study. The sample size and heterogeneity might have a potential impact on the divergent results, since only nine male ADHD patients and 11 healthy male volunteers were recruited in the O'Gorman's study.

In our study, the hypoperfusion areas in aADHD are primarily in the left hemisphere. Atypical lateralization has always been indicated in neuroimaging studies for ADHD.^42^, ^43^, ^44^ In line with our asymmetry results, previous studies found that altered CBF and functional connectivity existed in the left hemisphere among ADHD.^45^, ^46^ However, specific alterations in right hemisphere have also been reported in other studies.^47^, ^48^ In the future, more specialized and detailed exploration will help us to elucidate the defined pattern of lateralization in ADHD and promote our in‐depth understanding of its neuropathogenesis.

There is considerable evidence that ADHD involves dysfunctional modulation by the brain neurotransmitters dopamine (DA) and noradrenaline (NA) in frontal‐striatal network, which is part of frontoparietal network.^49^ CBF is modulated by the changes in neurotransmitters (eg, dopamine)^12^, ^50^ and nonspecific agents (eg, nitric oxide),^51^, ^52^ since these chemicals play a role in adjusting vascular response. One possible explanation for the hypoperfusion is that the abnormal dopamine system or nitric oxide system in ADHD may reduce the cerebral blood flow in certain brain regions. Abnormal dopamine system or nitric oxide system in ADHD may also contribute to abnormalities of vascular response.^50^, ^51^, ^52^ In the future, combination analyses with neurobiological (eg, NO/nNOS level) and genetic features (eg, *NOS1*‐ex1fVNTR) may help us to interpret the CBF abnormality in adults with ADHD more comprehensively.

### 4.2 Relationship between hypoperfusion and symptoms severity in adults with ADHD

We found a significant negative correlation between the CBF in the left amygdala/hippocampus and the total score. That means the more the CBF reduced, the higher the ADHD core symptoms indicated. In addition, a significant negative correlation existed between the CBF in the left putamen/global pallidum and the inattention score.

Correlation between CBF and the severity of ADHD core symptom has been barely reported in previous studies, most of which were obtained from functional connectivity‐based analyses. A previous study has demonstrated that inattention and hyperactivity/impulsivity symptom scores were positively correlated with functional connectivity in the networks of posterior putamen and ventral caudate.^53^ Another studies have shown that functional connectivity of the DMN and frontoparietal networks was modulated by the levels of inattention symptoms.^54^ Per Luca Cocchi et al^55^ found that the connectivity between the left amygdala and the right precentral gyrus in the ADHD group was negatively associated with the severity of past symptoms of inattention. The findings in current study were in consistent with previous studies that similar regions were indicated. Hippocampus, an important part of DMN for encoding the spatial and temporal relationships between sensory experiences and storage in long‐term memory, was crucial to the pathogenesis of ADHD. Based on these findings, it is reasonable to conjecture that hypoperfusion in the amygdala, hippocampus, and putamen may harm the region's capability to be recruited when responding to task demands. As a result, it might contribute to the impaired cognitive performance and ADHD symptoms. The spatial overlap of the ADHD‐related brain regions showing functional alterations between our current CBF‐based and previous FC‐based findings supported the neurovascular coupling in some extent. This also propelled us to further explore whether those brain regions with altered CBFs also indicated abnormality of functional connectivity.

### 4.3 Functional connectivity disruptions in the regions with hypoperfusion

Seed‐based functional connectivity analysis revealed that, compared to the healthy controls, left amygdala showed significantly decreased connectivity with PFC including superior frontal gyrus and left medial frontal gyrus, and supplementary motor area in adults with ADHD.

The amygdala plays an important role in emotional and social behavior, in charge of emotional processing.^56^ PFC is of vital importance for the emotional processing in “top‐down” regulation, while amygdala is involved in “bottom‐up” emotional processing.^57^, ^58^ Abnormal connectivities of amygdala with other regions spread across the lateral PFC, medial frontal region, rostral ACC, and right precentral gyrus, caudate nucleus, as well as the left orbitofrontal cortex in ADHD patients have been reported in the existing literature.^55^, ^59^, ^60^, ^61^ Emotional dysregulation is a commonly described feature of ADHD, even it has been suggested to be included in the diagnostic criteria as core components, especially for adults with ADHD.^62^ Our current finding might provide imaging evidence supporting the consideration of emotional symptoms in ADHD evaluation and diagnosis in the future. Considering the pathophysiological mechanisms of emotion‐related disorders were closely related to amygdala, we excluded all the ADHD patients with emotion‐related disorders for validation analysis. However, the results did not change substantially (Figures S4‐S7). The supplementary motor area is thought to be an important part of the core response inhibition system.^63^ In a meta‐analysis, superior/middle frontal gyrus and supplementary motor area are decreased brain activation in previous study.^64^ The findings in current study indicated the abnormalities in similar regions, which are overlapping with previous study.

The results in our study indicated that the regions with hypoperfusion in adults with ADHD showed potential functional connectivity alterations. Since BOLD signal is considered to measure the concentration of oxygen from hemoglobin in the brain, the hypoperfusion would change the local deoxyhemoglobin concentration in the brain, which might result in the changes of BOLD signal intensity and the functional connectivity of the whole brain.^65^ Meanwhile, the association between neuronal activity and blood perfusion might be bidirectional. The stronger functional connectivity tends to need greater metabolic demand, resulting in increased perfusion.^52^ The regulation of CBF in brain activity process involves the interaction of neurons, glia, and vascular cells.^66^ When the functional dysconnectivity exists, the neurons and glia could not generate the signals regulating the vascular changes. Thus, it might lead to hypoperfusion in ADHD. To establish the direct links between micro‐circulation and brain activity, we also have tried to perform correlation analyses between the CBF and rsFC for the ADHD‐related brain regions; however, no significant results were yielded (Table S3). In addition, we also did not find the correlation between abnormal functional connectivity and ADHD core symptoms. Similar phenomenon has also been indicated in other studies,^10^, ^67^ which might be due to the potential sequential relationship between CBF and FC or the more complexity of brain activities which may be resulted from other neurobiological changes in addition to the blood perfusion. In other opinion, in spite of negative findings for direct links, there might be some potential indirect links existed. For example, nNOS‐derived NO has been indicated to influence both the cerebral blood perfusion and monoaminergic transmitter systems^68^ that it might drive the indirect links between CBF and FC very likely. As we mentioned above, future combination analyses with neurobiological (eg, NO/nNOS level) and genetic features (eg, *NOS1*‐ex1fVNTR) may help us to verify this intriguing hypothesis. From another aspect, it might be suggested that CBF should be more sensitive as a more meaningful biomarker for the neuropsychiatric disorders.^67^

### 4.4 Limitations

There are several issues that should be further considered. Firstly, our current study only recruited male subjects to reduce the potential confounding effect of sample heterogeneity; however, this may also limit the generalizability of current findings to females. Secondly, the participants in current study are without the ADHD‐HI subtype. Finally, the IQs of both groups were high, although no significant differences were found in aADHD and healthy controls. Therefore, the findings in the study should be explained more cautiously and the further replications in samples with females, all subtypes, and general IQs are needed.

## 5 CONCLUSIONS

By combination of CBF and RSFCs, we found the alternations of cerebral perfusion and functional abnormality of the left amygdala associated with adults with ADHD. The combination of multimodal fMRI, such as ASL and BOLD, may provide complementary information and comprehensive insights to the pathophysiological mechanisms of ADHD from the perspective of combination of neuron and vessels.

## CONFLICT OF INTEREST

The authors declare no conflict of interest.
