# Supplementary material for: Alterations of cerebral perfusion and functional brain connectivity in medication‐naïve male adults with attention‐deficit/hyperactivity disorder
Source: CNS Neurosci Ther. 2019 Jun 23;26(2):197–206. doi: 10.1111/cns.13185 (PMC6978256; doi:10.1111/cns.13185)
Supplement: Supplementary file 1 [file CNS-26-197-s001.docx]

**Supplementary Materials**

None was excluded because of the excessive head movement

3 males with ADHD were excluded because of comorbidities:

Bipolar Disorder (N = 2)

Major Depressive Disorder (N = 1)

**Clinical Assessment**

ADHD RS-IV

A-WISC (IQ)

**MRI Experiment**

69 males with ADHD

69 healthy controls

3 patients with ADHD were excluded because of pharmacological therapies:

OROS-MPH (N = 3)

69 age-matched and IQ-matched healthy males from local healthy volunteers

72 males with ADHD

75 males with ADHD from psychiatric clinics

**Data Preprocessing**

**Further Analyses**

69 males with ADHD

69 healthy controls

**Figure S1:** Flowchart of recruitment process

**Table S1. Hypoperfused regions classified in seven functional network and AAL template**

| **7 Network from Yeo** | **Hypoperfused regions in aADHD** | | **Number of voxels** |  |
| --- | --- | --- | --- | --- |
| somatomotor network | | superior temporal gyrus, rolandic operculum, insula, supramarginal gyrus | 207 | |
| ventral attention network | | superior temporal gyrus, rolandic operculum, insula, supramarginal gyrus,middle temporal gyrus | 157 | |
| limbic network | | parahippocampal gyrus, amygdala, insula, inferior | 24 | |
| **AAL template** | |  |  | |
| sub-cortical regions | | insula, putamen, pallidam, amygdala, hippocampus, parahippocampal gyrus | 382 | |

ADHD, attention-deficit/hyperactivity disorder;

**Table S2. Correlations between amygdala-based rsFC and ADHD Symptoms**

| **Regions [*r* (*p*)]** | **Inattention** | **Hyperactivity/**  **impulsivity** | | **Total** |
| --- | --- | --- | --- | --- |
| **Left middle frontal gyrus**  **Left supplementary motor area**  **Right supplementary motor area**  **Left superior frontal gyrus**  **Right superior frontal gyrus** | 0.058 (0.638)  -0.093 (0.453)  0.087 (0.483)  -0.121 (0.328)  0.037 (0.767) | -0.118 (0.340)  -0.030 (0.807)  -0.004 (0.977)  -0.055 (0.658)  -0.076 (0.541) | -0.072 (0.565)  -0.097 (0.437)  0.014 (0.911)  -0.134 (0.281)  -0.040 (0.751) | |

ADHD, attention-deficit/hyperactivity disorder; rsFC: resting-state functional connectivity

**Table S3. Correlations between FCS and CBF**

| **Regions [*r* (*p*)]** | **CBF-FCS correlation** | |
| --- | --- | --- |
|  | **ADHD** | **HC** |
| **Left Putamen**  **Left Pallidum**  **Left Hippocampus**  **Left Amygdala** | -0.021 (0.862)  -0.032 (0.792)  0.054 (0.659)  0.066 (0.590) | -0.001 (0.993)  -0.038 (0.759)  0.079 (0.516)  0.048 (0.694) |

ADHD, attention-deficit/hyperactivity disorder; HC, healthy control; CBF, cerebral blood flow; FCS, functional connectivity strength

**
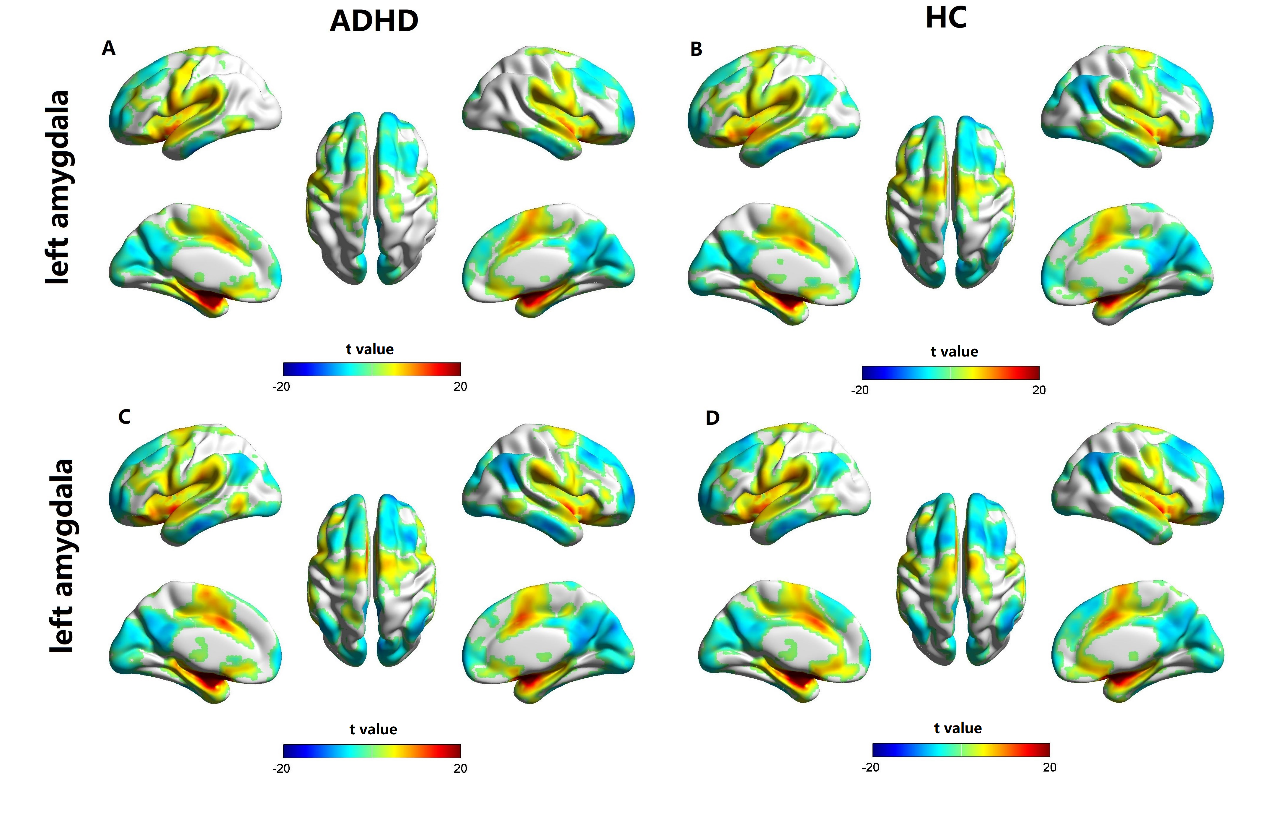
 Figure S2. Seed-based resting-state functional connectivity (RSFC) patterns of the left amygdala (hypoperfusion region) in the attention deficit/hyperactivity disorder (ADHD) and healthy control (HC) groups in the conditions of without (A and B) and with (C and D) scrubbing.** The yellow-red colors indicate that the brain regions were positively correlated with the seed ROI and the blue colors indicate that the brain regions were negatively correlated with the seed ROI.

**
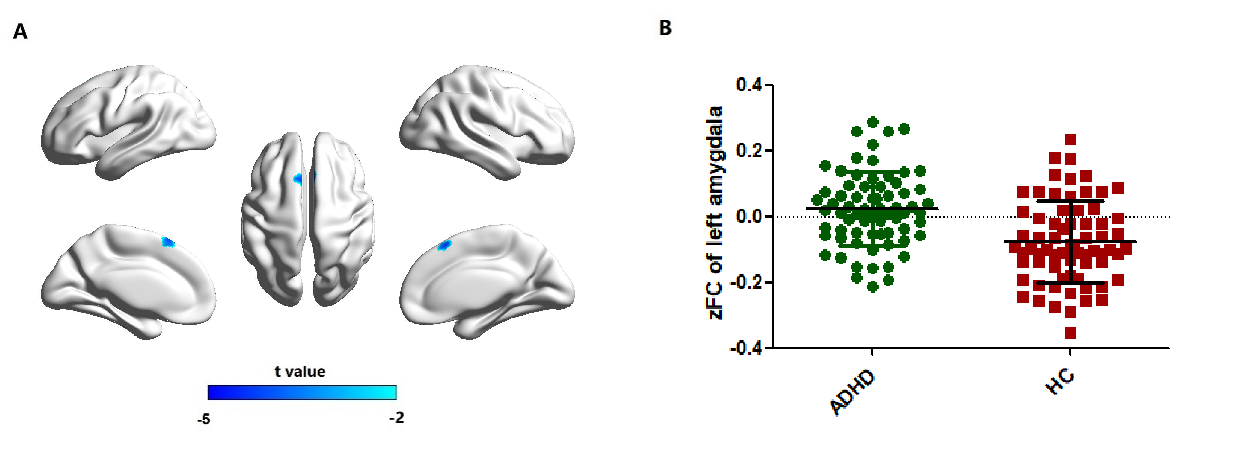
 Figure S3. Between-group comparisons for the seed-based functional connectivity with the seeds of left amygdala with scrubbing in the data preprocessing.** The independent two sample t-test was conducted between the ADHD group and the healthy control group. All results were corrected for multiple comparisons to a significant level of *p*<0.05 through a non-parametric permutation test (10,000 permutations, individual voxel *p*<0.01). FC, functional connectivity.


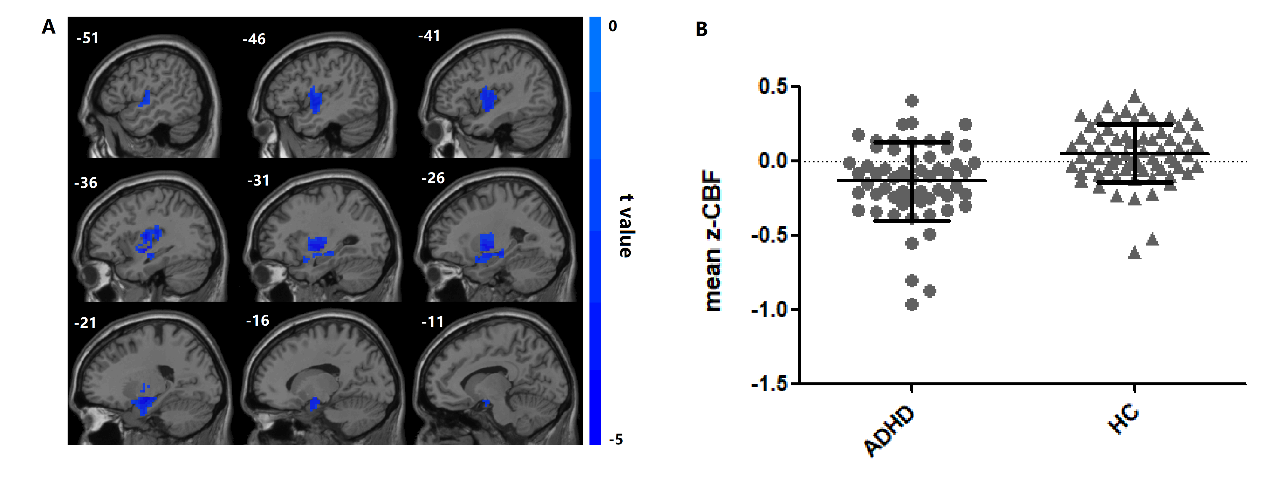
 **Figure S4. Group differences in CBF between ADHD patients without any emotion-related disorder and healthy controls.** The independent two sample t-test was conducted between the ADHD group and the healthy control group. All results were corrected for multiple comparisons to a significant level of *p*<0.05 through a non-parametric permutation test (10,000 permutations, individual voxel *p*<0.01) .The cold colors denote significantly decreased CBF in the ADHD patients. CBF, cerebral blood flow; HC, healthy controls; ADHD: attention-deficit/hyperactivity disorder


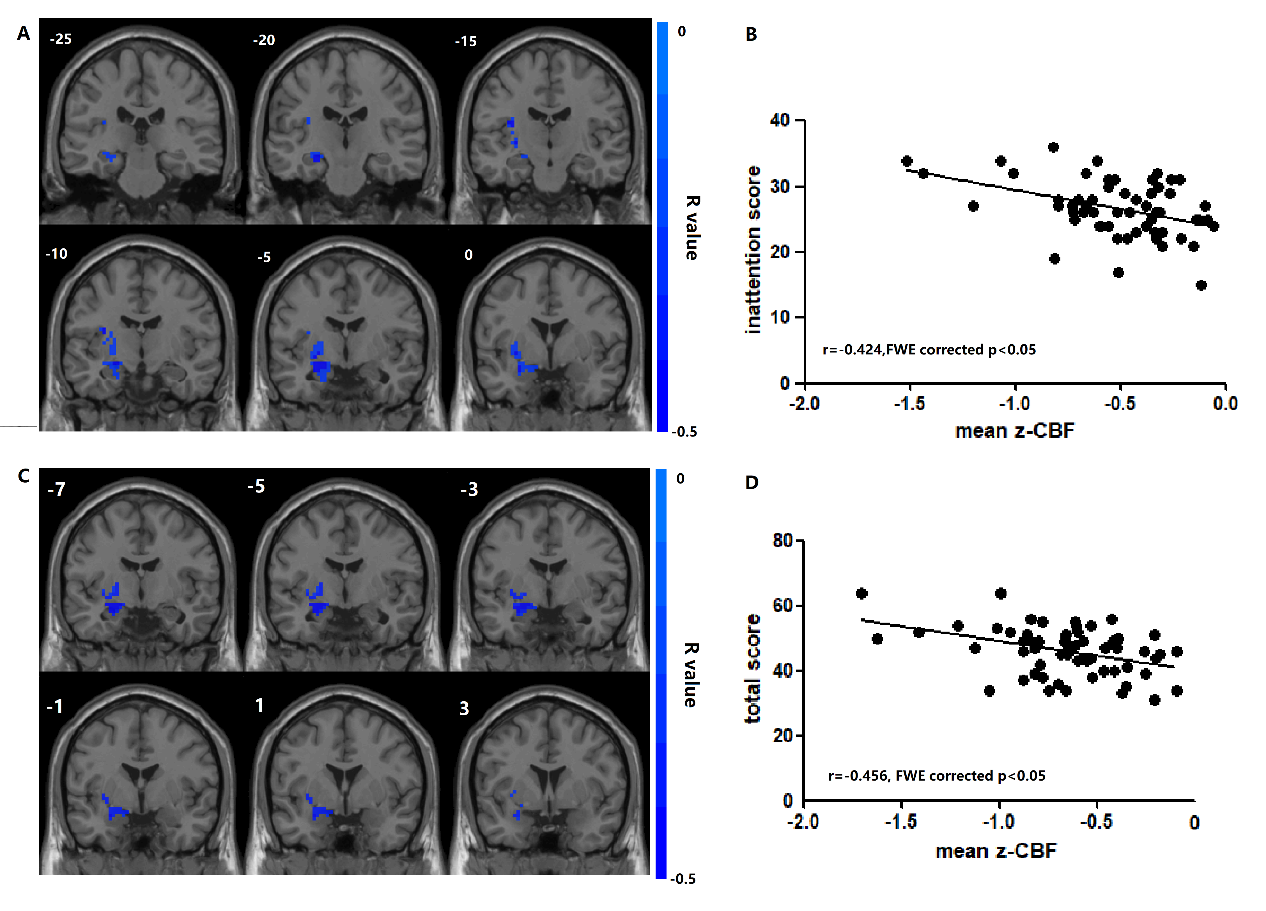


**Figure S5. The correlation between regions of the group differences (ADHD without any emotion-related disorder vs. HC) in CBF and ADHD core symptoms.** All results were corrected for multiple comparisons to a significant level of *p*<0.05 through a non-parametric permutation test (10,000 permutations, individual voxel *p*<0.01) A. The correlation between the left putamen/global pallidum/ amygdala/hippocampus/insular z-CBF and inattention scores in male aADHD. B. Scatter plot of correlation between the mean z-CBF of left putamen/global pallidum/ amygdala/hippocampus/insular and inattention scores in male aADHD. C. The correlation between the left putamen/global pallidum/ amygdala/hippocampus z-CBF and inattention scores in male aADHD. D. Scatter plot of correlation between the mean z-CBF of left putamen/global pallidum/ amygdala/hippocampus and total scores in male aADHD. CBF, cerebral blood flow. CBF, cerebral blood flow.


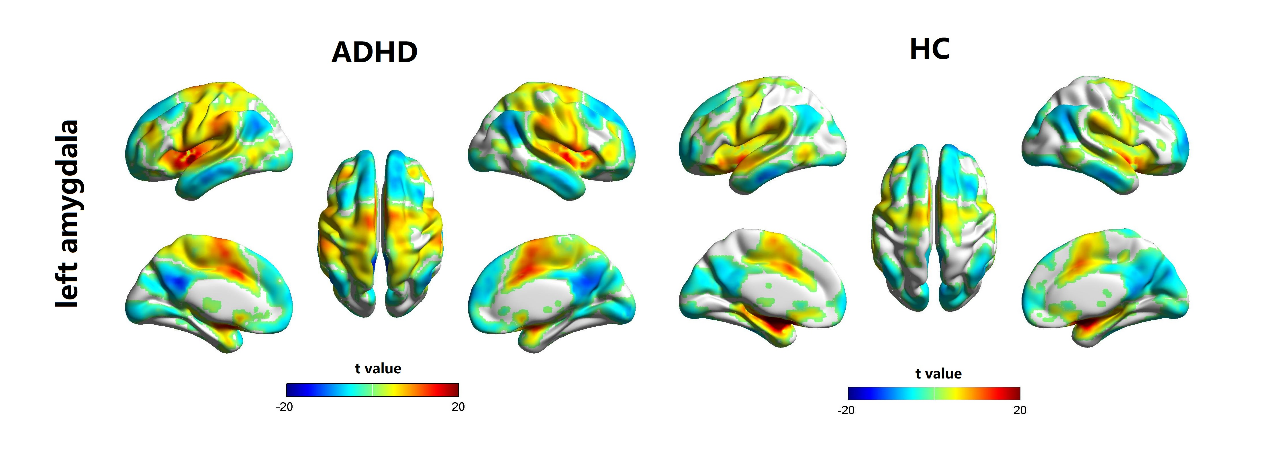


**Figure S6. Seed-based resting-state functional connectivity (RSFC) patterns of the left amygdala (hypoperfusion region) of ADHD without any emotion-related disorder and healthy control (HC) groups.** The yellow-red colors indicate that the brain regions were positively correlated with the ROI and the blue colors indicate that the brain regions were negatively correlated with ROI.


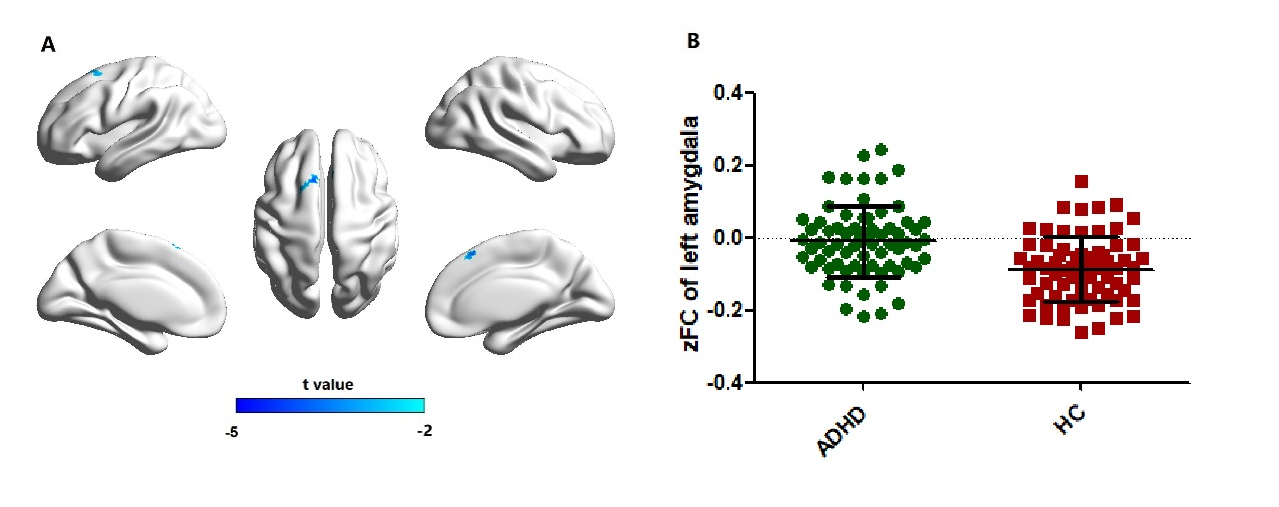


**Figure S7. Comparisons of the seed-based functional connectivity for left amygdala between ADHD without any emotion-related disorder and healthy controls.** All results were corrected for multiple comparisons to a significant level of *p*<0.05 through a non-parametric permutation test (10,000 permutations, individual voxel *p*<0.01). FC, functional connectivity.
